# Iron-Based Biochar for Efficient Persulfate Activation and Sulfamethoxazole Degradation

**DOI:** 10.3390/ijms26209971

**Published:** 2025-10-14

**Authors:** Ying Lu, Chengdu Qi, Guilong Peng, Yi Gao, Ronglong Zhang

**Affiliations:** 1 School of Mechatronics and Information Engineering, Chongqing College of Humanities, Science and Technology, Chongqing 401524, China; luyingcd2007@163.com; 2 State Key Laboratory of Resource Insects, College of Sericulture, Textile and Biomass Sciences, Southwest University, Chongqing 400715, China; pengguilong@swu.edu.cn; 3 School of Environment, Jiangsu Province Engineering Research Center of Environmental Risk Prevention and Emergency Response Technology, Jiangsu Engineering Lab of Water and Soil Eco-Remediation, Nanjing Normal University, Nanjing 210023, China; 4 College of Chemistry and Chemical Engineering, Southwest University, Chongqing 400716, China; hxraining@swu.edu.cn (Y.G.); zhangronglong@ccdri.cn (R.Z.)

**Keywords:** iron-loaded biochar, mulberry branches, persulfate, degradation, sulfamethoxazole

## Abstract

In this study, we investigated the performance of iron-loaded biochar (Fe-BC) derived from mulberry branches in activating persulfate (PS) for the efficient degradation of sulfamethoxazole (SMX). The Fe-BC/PS system exhibited superior catalytic activity towards SMX degradation, achieving 97% removal within 60 min. The degradation efficiency was found to be highly dependent on preparation conditions, including calcination temperature, the type of iron salt, and biomass feedstock. Reactive species such as hydroxyl radicals (^•^OH), sulfate radicals (SO_4_^•−^), and iron (IV) (Fe(IV)) were identified as key contributors to SMX degradation, with Fe(IV) playing a dominant role. The influence of water quality parameters, such as inorganic ions, pH, and natural organic matter (NOM), on the degradation of SMX was also examined. Proposed degradation pathways revealed the stepwise oxidation of SMX into smaller intermediates, ultimately leading to mineralization. Our findings highlight the potential of Fe-BC/PS systems as a sustainable and effective approach for the remediation of sulfonamide antibiotics in aquatic environments.

## 1. Introduction

Sulfonamide antibiotics, such as sulfamethoxazole (SMX), are widely used in human and veterinary medicine, leading to their frequent detection in various aquatic environments [1]. The presence of these antibiotics in water bodies poses a significant threat to aquatic ecosystems and human health, as they can bioaccumulate and cause long-term environmental and health issues [2]. Advanced oxidation processes (AOPs), particularly those involving persulfate (PS) activation, have emerged as promising technologies for the efficient degradation of organic pollutants, such as sulfonamide antibiotics [3,4]. PS, as a strong oxidant, can generate highly reactive sulfate radicals (SO_4_^•−^) upon activation, which exhibit high oxidation potential and selectivity towards multiple categories of environmental pollutants [5,6].

Various methods have been explored to activate PS, including thermal activation [7,8], ultraviolet (UV) irradiation [9], transition metal ions [10,11,12], and carbon-based materials [13]. Among the various PS activators, iron-loaded biochar (Fe-BC) attracted considerable attention due to its high surface area, abundant pore structure, and excellent catalytic properties [14,15]. Fe-BC, derived from the pyrolysis of iron-containing biomass, not only provides a sustainable source of iron for PS activation but also offers enhanced sorption capacities and catalytic activities, making it superior to traditional activators in terms of efficiency, stability, and environmental compatibility [16,17,18]. The synergistic combination of biochar and iron oxides demonstrates enhanced persulfate activation efficiency, generating multiple reactive species including hydroxyl radicals (^•^OH), sulfate radicals (SO_4_^•−^), and iron(IV) (Fe(IV)), which are potent oxidants capable of degrading diverse organic pollutants [19].

In the present study, we aimed to investigate the performance of Fe-BC derived from agricultural waste, specifically mulberry branches, in activating PS for the efficient degradation of SMX. This approach embodies the concept of “waste-to-wealth”, utilizing waste biomass to produce a valuable catalyst for environmental remediation. The crystalline structure and surface morphology of Fe-BC were characterized using X-ray diffraction (XRD) and scanning electron microscopy (SEM), respectively, to gain insights into its physical and chemical properties. We also examined the effect of various preparation conditions, including calcination temperature, iron loading, and biomass feedstock, on the catalytic activity of Fe-BC. Furthermore, we investigated the influence of the key water quality parameters, including inorganic ions, pH, and natural organic matter (NOM), on SMX degradation to understand the practical applicability of our Fe-BC/PS system.

To gain deeper insights into the mechanisms underlying SMX degradation, we conducted quantitative and qualitative analyses of the reactive species involved in the Fe-BC/PS system. Specifically, we determined the steady-state concentrations of ^•^OH, SO_4_^•−^, and Fe(IV) using competition kinetics experiments and calculated their respective contributions to SMX degradation. These findings not only provide valuable insights into the mechanisms of SMX degradation in Fe-BC/PS systems but also highlight the potential of this technology for the remediation of sulfonamide antibiotics in aquatic environments. The utilization of agricultural waste as a feedstock for Fe-BC production further underscores the sustainability and environmental benefits of our approach.

## 2. Results and Discussion

### 2.1. Characterizations

**Figure 1:**
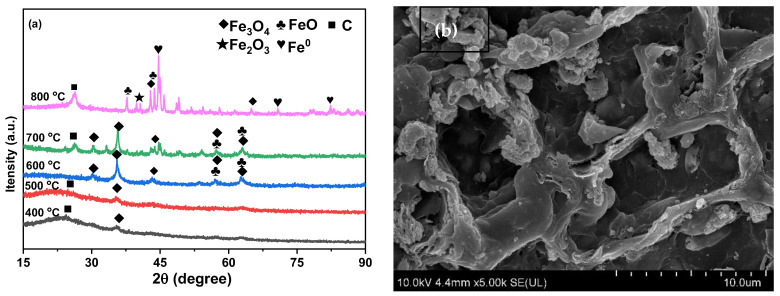
(**a**) XRD and (**b**) SEM of the Fe-BC.

The XRD patterns presented in Figure 1a reveal specific crystallographic features of the Fe-BC samples prepared at various calcination temperatures. A notable weak peak at 35.83° is observed for the Fe-BC synthesized at both 400 °C and 500 °C, which can be attributed to the presence of Fe_3_O_4_ [17]. When the calcination temperature is raised to 600 °C and 700 °C, the XRD spectra exhibit peaks corresponding to both Fe_3_O_4_ and Fe_2_O_3_ at 35.83°, 57.11°, and 62.72° [20]. Upon further increasing the calcination temperature to 800 °C, a significant decrease or complete disappearance of the Fe_3_O_4_ and Fe_2_O_3_ peaks is evident. These peaks are subsequently replaced by Fe^0^, appearing at 44.63°, 65.11°, and 82.52° [21], suggesting a transformation in the iron oxide phases. The SEM photos of the Fe-BC are shown in Figure 1b; the image reveals a complex network of porous structures with varying sizes and shapes, indicative of the material’s high surface area and potential for applications in catalysis and environmental remediation.

### 2.2. Catalytic Removal of SMX in Different Systems

**Figure 2:**
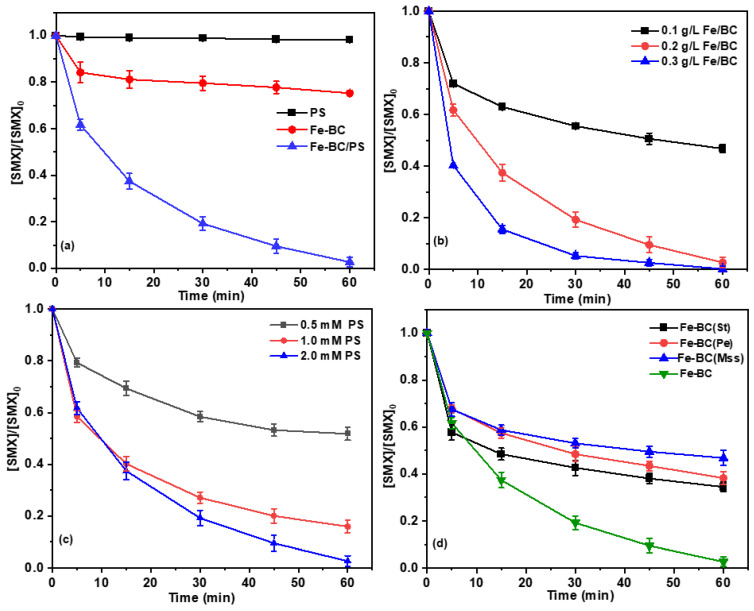
(**a**) Degradation kinetics of SMX in different systems; effect of (**b**) Fe-BC dosage, (**c**) PS dosage, (**d**) different biomass feedstocks on SMX removal efficiencies in the Fe-BC/PS system (Conditions: except for the investigated parameter, the other parameters were fixed at: [Fe-BC]_0_ = 0.2 g/L, [PS]_0_ = 2.0 mM, [SMX]_0_ = 10 mg/L, pH_0_ = 3.1, T = 25 °C).

The removal efficiencies of SMX in PS, Fe-BC, and Fe-BC/PS systems were investigated. As can be seen from Figure 2a, less than 2% of SMX (10 mg/L) was removed within 60 min in a separate PS (2.0 mM) system, suggesting that the oxidative capability of PS towards SMX can be neglected. Meanwhile, the removal of SMX was less than 25% when only Fe-BC (0.2 g/L) was added, indicating that the removal of SMX by adsorption was limited. However, a significant degradation efficiency of 97% of SMX can be achieved in the Fe-BC/PS system, highlighting the important role of the catalyst in improving PS activation. The chromatogram (Figure S1a) depicted a progressive diminution in the signal intensity corresponding to SMX over a period of 60 min. This temporal trend indicated a steady degradation process in the Fe-BC/PS system. Moreover, the concentration of iron leached during the reaction remained consistently below 2.5 mg/L (2.03 mg/L at 5 min and 2.42 mg/L at 60 min, Figure S1b), indicating that the Fe-BC catalyst exhibited low iron leaching, which highlights its good stability and environmental compatibility. In addition, the total organic carbon (TOC) removal efficiency increased from about 20% at 5 min to 35% at 60 min (Figure S1c), indicating that SMX was mainly oxidized into smaller intermediates rather than being fully mineralized. These results collectively demonstrate that the Fe-BC/PS system not only exhibited excellent catalytic activity but also maintained good stability with limited iron leaching.

The Fe-BC/PS system operates through synergistic interaction between iron-modified biochar (Fe-BC) and persulfate (PS). Systematic evaluation of catalyst loading effects revealed that SMX degradation exhibited dose dependency within 0.1–0.3 g/L Fe-BC (Figure 2b). Quantification analysis demonstrated a near-linear enhancement in contaminant removal efficiency from 53% to 97% (ΔR = +44%) across the tested concentration range. These enhancements could be primarily ascribed to the increased amount of active sites on Fe-BC surface, which facilitates both SMX adsorption and PS activation as the dosage of Fe-BC is increased. This, in turn, results in the production of more reactive species and achieves more efficient SMX degradation [17,22]. Meanwhile, the observed rate constants (*k_obs_*) for SMX degradation increased from 1.1 × 10^−2^ min^−1^ to 9.4 × 10^−2^ min^−1^ with the increase in Fe-BC dosage from 0.1 g/L to 0.3 g/L, further confirming the critical role of catalyst dosage in optimizing reaction kinetics. Meanwhile, the observed rate constants (*k_obs_*) for SMX degradation rose from 1.1 × 10^−2^ min^−1^ to 9.4 × 10^−2^ min^−1^ as Fe-BC concentration increased from 0.1 g/L to 0.3 g/L, further confirming the critical role of catalyst dosage in optimizing reaction kinetics. These results underscore the feasibility of tailoring Fe-BC loading to achieve cost-effective and high-performance degradation of sulfonamide antibiotics in water treatment systems.

In addition, the removal of SMX by PS activated with Fe-BC was evaluated at different concentrations of PS (0.5, 1, and 2 mM). As depicted in Figure 2c, the removal of SMX increased significantly with higher PS concentrations. Within 60 min, the degradation efficiencies of SMX reached 48%, 84%, and 97% for PS concentrations of 0.5, 1, and 2 mM, respectively. The *k_obs_* for SMX degradation increased from 1.0 × 10^−2^ to 5.6 × 10^−2^ min^−1^ as the PS dosage increased from 0.5 to 2.0 mM. This trend may imply that the generation of reactive species increased with higher PS concentrations, leading to enhanced degradation of SMX [23].

Furthermore, the results of PS activation by Fe-BC derived from different biomass feedstocks for SMX degradation are presented in Figure 2d. Among the tested catalysts, Fe-BC(Mu) prepared from mulberry branches demonstrated superior catalytic performance, achieving 97% SMX degradation within 60 min. In contrast, Fe-BC(Pe), Fe-BC(St), and Fe-BC(Mss), derived from peanut shells, rice straw, and melon seed shells, respectively, exhibited moderate degradation efficiencies ranging between 53 and 66% under identical reaction conditions. This significant performance variation among different biomass-derived catalysts could be attributed to the distinct cellulose contents in the precursor materials. The higher cellulose content in mulberry branches likely contributed to the formation of a more porous carbon structure with enhanced iron dispersion during pyrolysis, thereby facilitating more effective persulfate activation and pollutant degradation [24].

### 2.3. Effect of the Material Preparation Conditions on the Degradation of SMX

**Figure 3:**
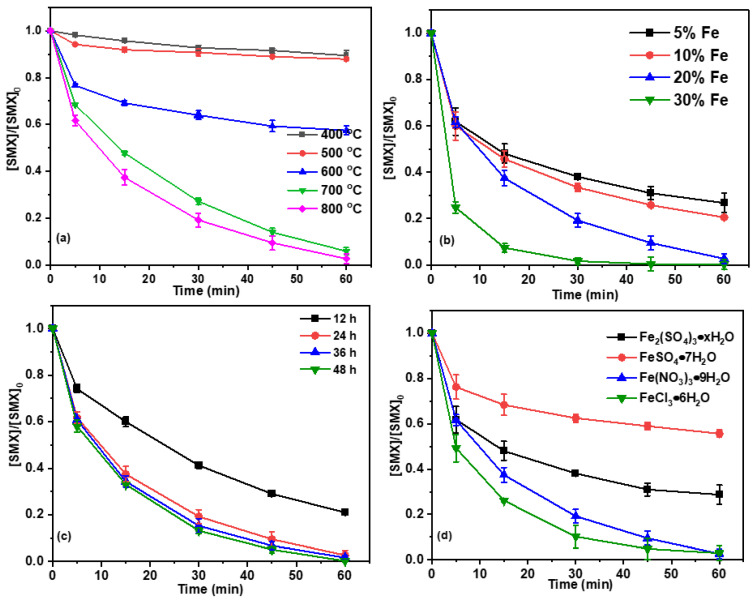
Effect of (**a**) calcination temperatures, (**b**) Fe contents, (**c**) soaking duration and (**d**) different iron salts on SMX removal efficiencies in the Fe-C/PS system (Conditions: [Fe-BC]_0_ = 0.2 g/L, [PS]_0_ = 2.0 mM, [SMX]_0_ = 10 mg/L, pH_0_ = 3.1, T = 25 °C).

The impact of calcination temperature (400–800 °C) on Fe-BC-mediated PS activation for SMX elimination is depicted in Figure 3a. These results indicated that the removal of SMX was markedly improved with increasing calcination temperature. Specifically, the SMX removal rate was only 15% for Fe-BC calcined at 400 °C, while it increased to 95% and 97% for those calcined at 700 °C and 800 °C, respectively. These results indicated the critical role of calcination temperature in determining the catalytic performance of Fe-BC in persulfate activation. The enhanced catalytic performance of Fe-BC at elevated calcination temperatures can be attributed to multiple synergistic effects. Higher temperatures promote more uniform dispersion of iron species throughout the biochar matrix, consequently creating additional active sites for persulfate activation [25]. Concurrently, elevated temperatures enhance the development of biochar’s porous structure and specific surface area, which not only improves SMX adsorption capacity but also facilitates interfacial interactions between persulfate and SMX molecules. Furthermore, high-temperature calcination may induce favorable redox cycling of iron species, which enhances their catalytic activity [26,27]. The optimal catalytic performance observed at 800 °C suggests that this temperature achieves an ideal balance between iron speciation and biochar structural properties, thereby maximizing both persulfate activation efficiency and SMX degradation.

Figure 3b revealed the degradation kinetics of SMX using Fe-BC activated PS under varying Fe contents. The experiments were conducted with Fe loadings of 5%, 10%, 20%, and 30% (*w*/*w*) on the biochar surface, and the reaction was monitored over a period of 60 min. The results showed that the removal efficiency of SMX increased progressively with the increase in Fe content. Specifically, the degradation efficiencies of SMX were 73%, 79%, and 97% for Fe loadings of 5%, 10%, and 20%, respectively, within 60 min. It is noteworthy that Fe-BC prepared with a 30% iron loading can achieve nearly complete degradation of SMX within just 30 min. The enhanced degradation efficiency of SMX with increasing Fe content could be attributed to the higher availability of catalytic active sites on the biochar surface. As the Fe loading increased, the amount of active sites for PS activation also increased, leading to the generation of a greater amount of reactive species [28]. Additionally, the increased Fe content may have facilitated electron transfer on the biochar surface, thereby accelerating the overall degradation process [19].

The influence of soaking duration of the mixed Fe(NO_3_)_3_•9H_2_O and mulberry branch powder on the performance of the prepared Fe-BC in activating PS for the degradation of SMX was also investigated. Fe-BC was prepared from a mixture of Fe(NO_3_)_3_•9H_2_O and mulberry branch powder that had been soaked in water for different durations (12, 24, 36, and 48 h) followed by drying. The removal of SMX was then evaluated using these Fe-BC. As shown in Figure 3c, the removal efficiency of SMX increased with the soaking duration. Specifically, the SMX degradation efficiencies were 79% for the biochar prepared from the mixture soaked for 12 h, and 97%, 98%, and 98% for those soaked for 24, 36, and 48 h, respectively. These results indicate that soaking the mixture for 24 h was sufficient to achieve a high degradation efficiency of SMX, with further increases in soaking time (36 and 48 h) resulting in only marginal improvements in removal rates. Therefore, a soaking duration of 24 h was considered optimal for preparing the Fe-based biochar for efficient PS activation and SMX degradation in this study.

Additionally, different iron salts to prepare Fe-BC on the performance of Fe-BC in activating PS for the degradation of SMX was investigated (Figure 3d). Fe-BC samples were prepared using mulberry branch powder combined with various iron salts, including Fe_2_(SO_4_)_3_, FeSO_4_, Fe(NO_3_)_3_, and FeCl_3_. The results showed significant differences in SMX degradation efficiency among the different Fe-BC samples. Specifically, the degradation efficiencies of SMX were 71.26% for Fe-BC prepared with Fe_2_(SO_4_)_3_, 44% for FeSO_4_, and 97% for Fe(NO_3_)_3_. Notably, Fe-BC prepared with FeCl_3_ achieved nearly 100% SMX degradation within 30 min, indicating superior performance compared to other iron salts. This suggests that the choice of iron salt significantly impacts the catalytic activity of Fe-BC in activating PS for SMX degradation, with FeCl_3_ being the most effective under the tested conditions. These findings highlight the importance of selecting appropriate iron salts during Fe-BC synthesis to optimize the degradation efficiency of SMX through PS activation.

### 2.4. Identification of Reactive Species

**Figure 4:**
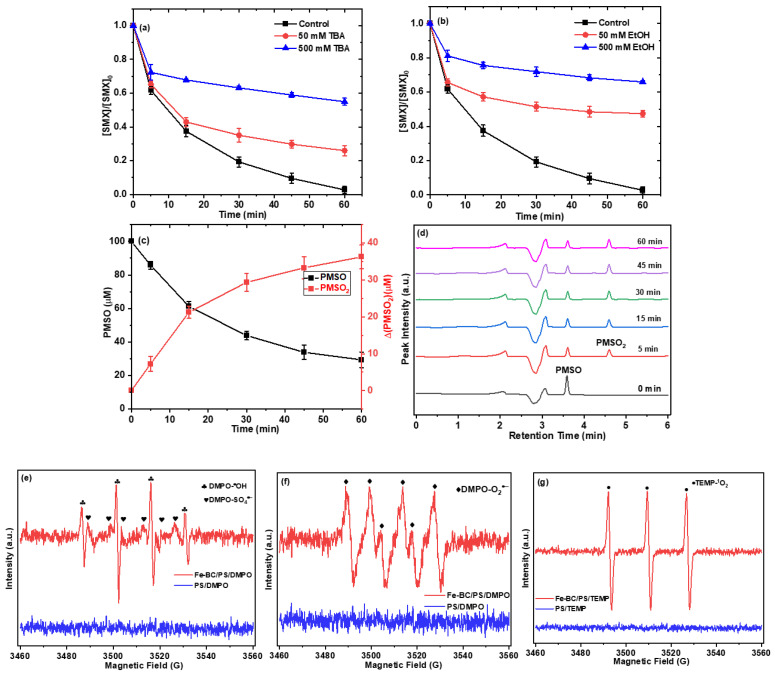
Effect of (**a**) TBA and (**b**) EtOH on SMX removal efficiencies; (**c**) oxidation of PMSO and production of PMSO_2_; (**d**) HPLC chromatogram recorded for the oxidation of PMSO and production of PMSO_2_; EPR measurements of (**e**) ^●^OH and SO_4_^●−^, (**f**) O_2_^•−^ and (**g**) ^1^O_2_. (Conditions: [Fe-BC]_0_ = 0.2 g/L, [PS]_0_ = 2.0 mM, [SMX]_0_ = 10 mg/L, [PMSO]_0_ = 100 μM, pH_0_ = 3.1, T = 25 °C. Note: The detection of ^•^OH and SO_4_^•−^ (**e**) was conducted in aqueous solution, O_2_^•−^ detection (**f**) was carried out in methanol to suppress ^•^OH and SO_4_^•−^ signals, and ^1^O_2_ detection (**g**) was performed in aqueous solution).

To identify the reactive radical species involved in the present reaction system, quenching experiments were conducted using various chemical quenchers. Ethanol (EtOH) is known to effectively scavenge both ^•^OH and SO_4_^•−^ radicals (k″OH•,EtOH=2.8×109 M−1s−1 and ${k^{\prime \prime }}_{SO_{4}^{•-},EtOH}=7.7\times 10^{7}\;M^{-1}s^{-1}$) [29], while tert-butyl alcohol (TBA) preferentially scavenges ^•^OH (k″OH•,TBA=6.0×108 M−1s−1) but is less effective against SO_4_^•−^ (${k^{\prime \prime }}_{SO_{4}^{•-},TBA}=4.0\times 10^{5}\;M^{-1}s^{-1}$) [29]. As can be seen from Figure 4a,b, in the absence of a quencher, the control group without a quencher achieved 97% SMX removal within 60 min. The addition of 50 mM and 500 mM TBA reduced the degradation efficiency to 74% and 45%, respectively. Ethanol (EtOH) quenching tests revealed more significant suppression effects compared to TBA, with SMX degradation efficiency decreasing to 53% and 34% at identical concentrations (50 mM and 500 mM). The distinct quenching behaviors of TBA (effective for ^•^OH) and EtOH (effective for both ^•^OH and SO_4_^•−^) suggest that both ^•^OH and SO_4_^•−^ radicals played a vital role in the removal of SMX. Nevertheless, upon introducing FAA as a quencher for singlet oxygen (^1^O_2_) and *p*-BQ as a quencher for superoxide radicals (O_2_^•−^) into the reaction system [22], the degradation efficiency of SMX experienced a slight decline with the increase in p-BQ (0.1 to 1.0 mM) (Figure S2a) and FFA (1 mM to 10 mM) (Figure S3b).

Based on previous research suggesting the potential generation of Fe(IV) in the activation of persulfate by iron-based activated systems, we designed an experiment to investigate this possibility using PMSO as a probe compound. Fe(IV) is known to specifically transform PMSO into PMSO_2_, a product distinct from those formed by radical reactions. In our study, the degradation of PMSO in the Fe-BC/PS system was employed, and the depletion of PMSO was observed to be 70.6 μM, with a corresponding production of PMSO_2_ at 30.3 μM (Figure 4c,d). This finding indicates the presence of Fe(IV) in the Fe-BC/PS system. However, the yield of PMSO_2_ was only about 51% (Figure S4), significantly lower than the theoretically calculated maximum value of 100%. This reduced yield can be attributed to the presence of both ^•^OH and SO_4_^•−^ in the Fe-BC/PS system, which are highly reactive species that can also react with PMSO (kSO4•−,PMSO=3.61×109,kOH•,PMSO=3.17×108) [30]. These high reactivity rates explain the significant consumption of PMSO by these radicals, leading to the lower observed yield of PMSO_2_.

To further elucidate the reaction mechanisms in the Fe-BC/PS system, Electron Paramagnetic Resonance (EPR) analyses were performed using DMPO as a spin-trapping agent for radicals and TEMP as a selective trapping agent for singlet oxygen (^1O_2_). Such spin-trapping approaches are widely employed in persulfate-based AOP studies. As shown in Figure 4e–g, characteristic EPR signatures of multiple reactive oxygen species (ROS) were identified. Distinct signals corresponding to DMPO–^•^OH and DMPO–SO_4_^•−^ adducts (Figure 4e) confirmed the concurrent formation of hydroxyl and sulfate radicals. A typical spectrum with four main peaks and two shoulder peaks (Figure 4f) was attributed to the DMPO–O_2_^•−^ adduct, evidencing the generation of superoxide radicals. Moreover, the pronounced three-line signal of the TEMP–^1^O_2_ adduct (Figure 4g) indicated the presence of singlet oxygen.

Taken together, these observations demonstrate that Fe(IV), ^•^OH, SO_4_^•−^, O_2_^•−^, and ^1^O_2_ are all generated in the Fe-BC/PS system. The coexistence of both radical (^•^OH, SO_4_^•−^, O_2_^•−^) and non-radical (^1^O_2_, Fe(IV)) pathways provides direct mechanistic evidence for a diverse ROS profile, highlighting the synergistic contributions of biochar surface functionalities, iron species, and persulfate activation to the broad-spectrum oxidative performance of the system.

Based on both the present experimental data and relevant literature, we propose a plausible mechanism in which BC facilitates electron transfer to O_2_, thereby promoting the generation of O_2_^•−^ (Equation (1)) [31,32]. This electron transfer process is crucial, as it not only triggers the formation of additional ROS but also enhances the overall oxidative capacity of the system. Furthermore, the observed ^1^O_2_ signals can be attributed to the reactions outlined in Equations (2) and (3) [17], which describe pathways involving the interaction between persulfate and the Fe-BC catalyst.

Taken together, these results provide new insight into the complex mechanisms governing the decrease in SMX concentration in the Fe-BC/PS system, where multiple ROS and their dynamic interactions with both the catalyst and persulfate contribute to the overall reactivity.

(1) $$BC+O_{2}\to O_{2}^{•-}+BC^{+}$$

(2) O2•−+O2•−+H+→O21+H2O2

(3) OH•+O2•−→O21+OH−

### 2.5. Contribution of Different Reactive Species to SMX Degradation

**Figure 5:**
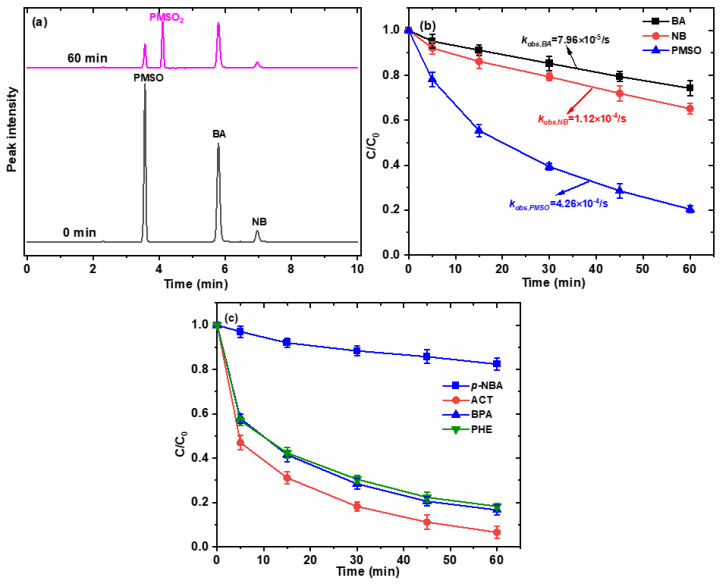
(**a**) HPLC chromatogram recorded for the mixed system containing NB, BA, and PMSO collected during treatment by the Fe-BC/PS system; (**b**) degradation kinetics for NB, BA, and PMSO; (**c**) degradation kinetics for different contaminants (Conditions: [Fe-BC]_0_ = 0.2 g/L, [PS]_0_ = 2.0 mM, [NB]_0_ = [BA]_0_ = 20 µM, [PMSO]_0_ = 100 µM, [contaminants]_0_ = 10 mg/L, pH_0_ = 3.1, T = 25 °C).

To gain deeper insights into the specific roles of ^•^OH, SO_4_^•−^, and Fe(IV) in the degradation of SMX, we employed a competition kinetics experiment to quantify their steady-state concentrations. This experiment was conducted in a mixed-probe system containing NB, BA, and PMSO, following our previously reported approach [24]. Representative chromatograms of the mixed probes at 0 and 60 min are shown in Figure 5a, which demonstrate effective separation of the compounds and the emergence of a new peak corresponding to PMSO_2_. These results confirm the validity of the mixed-probe method and ensure the reliability of the subsequent quantitative analysis. As can be seen from Figure 5a,b, during the reaction, the concentrations of PMSO, BA, and NB decrease. As illustrated in Figure 5b, the first-order rate constants (*k_obs_*) for BA, NB, and PMSO were determined to be 7.96 × 10^−5^/s, 1.12 × 10^−4^/s, and 4.26 × 10^−4^/s, respectively. Based on Equations (10), (11) and (13), the [^•^OH]_ss_, [SO_4_^•−^]_ss_, and [Fe(IV)]_ss_ were determined to be 2.87 × 10^−14^ M, 1.12 × 10^−14^ M, and 2.59 × 10^−9^ M, respectively. To further elucidate the role of these species in SMX degradation, their relative contributions were evaluated using Equations (15)–(18). The results revealed distinct contributions, with ^•^OH and SO_4_^•−^ accounting for 26% and 19% of SMX removal, respectively, while adsorption contributed 6%. In contrast, Fe(IV) dominated with a 49% contribution, highlighting its key role in SMX degradation. This discrepancy between Fe(IV)’s high steady-state concentration and its predominant role highlights its superior oxidative selectivity toward SMX. Specifically, Fe(IV) preferentially targets electron-donating functional groups (e.g., the aniline moiety in SMX) via non-radical electron transfer pathways, as opposed to the indiscriminate oxidation mediated by ^•^OH and SO_4_^•−^. The findings align with prior studies on high-valent iron species and underscore Fe(IV) as the pivotal driver of SMX degradation in this system [19,30].

To further substantiate the distinct reactivity of Fe(IV) compared to ^•^OH and SO_4_^•−^ towards different contaminants, we conducted a comparative degradation study under identical experimental conditions, examining the removal efficiencies of bisphenol A (BPA), paracetamol (acetaminophen, ACT), phenol (PHE), and *p*-nitrobenzoic acid (*p*-NBA). As depicted in Figure 5c, it is evident that BPA, ACT, and PHE exhibit remarkable degradation efficiencies, with more than 90% removal observed within 60 min. Specifically, the degradation curves for BPA, ACT, and PHE demonstrate steep declines, indicative of rapid and efficient oxidation processes. This pattern suggests that these compounds are highly susceptible to the oxidative species present in the system, particularly Fe(IV), given its dominant role as revealed earlier. In stark contrast, the degradation profile of *p*-NBA shows a significantly different trend. Unlike the other contaminants, *p*-NBA displays a much slower degradation rate, with only 18% degradation efficiency observed. This finding underscores *p*-NBA’s resistance to the oxidative species in the system, particularly Fe(IV), which is presumed to be the primary oxidant due to its high steady-state concentration and dominant contribution to SMX degradation. The observed difference in degradation efficiencies can be attributed to the structural and chemical properties of the contaminants. BPA, ACT, and PHE possess electron-donating functional groups that are susceptible to attack by Fe(IV) via non-radical electron transfer pathways, leading to their rapid degradation. Conversely, *p*-NBA’s nitro and carboxyl groups, which are electron-withdrawing, may confer stability against oxidation by Fe(IV) and other reactive species, resulting in its slower degradation rate. These results not only reinforce the pivotal role of Fe(IV) in the degradation of SMX but also extend our understanding to other contaminants, highlighting its selectivity towards electron-donating moieties.

### 2.6. Influence of Water Quality Parameters on the Degradation of SMX

**Figure 6:**
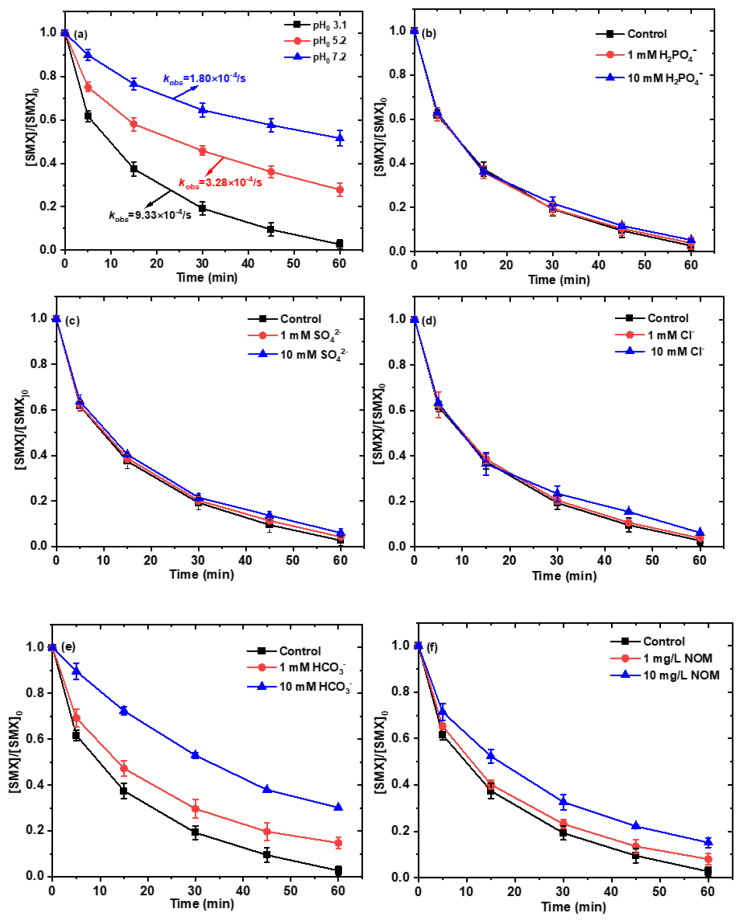
(**a**) Removal efficiencies of SMX at different initial pH values; (**b**) effect of H_2_PO_4_^−^, (**c**) SO_4_^2−^, (**d**) Cl^−^, (**e**) HCO_3_^−^ and (**f**) NOM on the removal of SMX (Conditions: [Fe-BC]_0_ = 0.2 g/L; [PS]_0_ = 2.0 mM; [SMX]_0_ = 10 mg/L; pH_0_ = 3.1 for panels (**b**–**d**,**f**), and pH_0_ = 8.4 for panel (**e**); T = 25 °C).

The initial pH plays an important role in the performance of iron-based biochar-activated persulfate for SMX degradation. To investigate the influence of pH on the activation performance in the Fe-BC/PS system, the degradation efficiency of SMX was examined at initial pH values of 3.1, 5.2, and 7.2 (Figure 6a). At an initial pH of 3.1, the degradation efficiency of SMX reached 97%, with an observed *k_obs_* of 5.60 × 10^−2^ min^−1^. As the initial pH increased to 5.2, the degradation efficiency decreased to 72%, accompanied by a reduction in the *k_obs_* to 1.97 × 10^−2^ min^−1^. Further increasing the initial pH to 7.2 resulted in a significant drop in degradation efficiency to 48%, with a corresponding *k_obs_* of 1.08 × 10^−2^ min^−1^. The decline in performance with increasing pH can be primarily ascribed to the reduced leaching of iron from Fe–BC and the consequent decrease in persulfate activation efficiency. Under acidic conditions, Fe(II) species are more readily released from the biochar, which efficiently activate persulfate to generate reactive species capable of rapidly degrading SMX. As the solution pH increases, iron leaching is suppressed, resulting in lower levels of soluble Fe(II) and diminished activation of persulfate. This directly leads to decreased generation of ^•^OH and SO_4_^•−^, as confirmed by the weaker EPR signals at higher pH values (Figure S5). In addition, hydroxide ions (OH^−^) at elevated pH compete with SMX for reactive species, acting as radical scavengers. This competitive effect further reduces the availability of ^•^OH and SO_4_^•−^ for SMX degradation. Collectively, these factors explain the strong dependence of SMX removal efficiency on the initial pH.

Inorganic ions are ubiquitous in aquatic environments and have the potential to substantially influence the oxidation of contaminants within the Fe-BC/PS system. Consequently, the impact of selected inorganic anions (specifically, H_2_PO_4_^−^, SO_4_^2−^, Cl^−^, and HCO_3_^−^) on the degradation of SMX by the Fe-BC/PS system was investigated. The experimental data presented in Figure 6b–e provide insights into the specific effects of H_2_PO_4_^−^, SO_4_^2−^, Cl^−^, and HCO_3_^−^ on the degradation of SMX in the Fe-BC/PS system. As evident from the results, H_2_PO_4_^−^ and SO_4_^2−^, exhibit minimal influence on the degradation of SMX. The presence of these anions cannot significantly alter the degradation efficiency, suggesting that they do not interfere with the oxidative process catalyzed by the Fe-BC/PS system to a great extent. Similarly, Cl^−^ also demonstrates negligible impact on the degradation of SMX. This suggests that Cl^−^ does not play a substantial role in either promoting or inhibiting the oxidation process in the reaction system. In contrast, HCO_3_^−^ displays a notable inhibitory effect on the degradation of SMX. This observation can be attributed to several factors. Firstly, the introduction of HCO_3_^−^ results in a significant increase in the solution’s pH to 8.4, creating an alkaline environment that is unfavorable for the activation of PS. Alkaline conditions are known to reduce the effectiveness of PS in generating reactive species, thereby hindering the degradation process, which was consistent with the experimental findings regarding the impact of initial solution pH. Secondly, HCO_3_^−^ has been shown to quench both ^•^OH and SO_4_^•−^, which are responsible for the degradation of SMX in the Fe-BC/PS system. The quenching of these radicals by HCO_3_^−^ leads to the formation of carbonate radicals (CO_3_^•−^, E^0^ = 1.78 V) (Equations (4) and (5)) [32], which are less oxidative than ^•^OH (E^0^ = 2.8 eV) and SO_4_^•−^ (E^0^ = 2.6–3.1 eV) [33]. Consequently, the overall degradation efficiency of SMX is compromised in the presence of HCO_3_^−^. Overall, the experimental data presented in Figure 6b–e highlight the differential impacts of various inorganic anions on the degradation of SMX in the Fe-BC/PS system, with HCO_3_^−^ emerging as a significant inhibitor due to its effects on solution pH and its ability to quench critical oxidative species.

Natural organic matter (NOM), ubiquitously present in aquatic environments, has been extensively documented for its remarkable capacity to interact with free radicals, consequently exerting significant influence on the degradation kinetics and removal efficiency of target contaminants during AOPs [34]. Given this background, the current study investigated the effect of humic acid (HA), specifically in the form of sodium humate, on the degradation of sulfamethoxazole (SMX) in the Fe-BC/PS system. Two concentrations of NOM, 1 mg/L and 10 mg/L, were introduced into the reaction system to assess their impact on SMX degradation. As illustrated in Figure 6f, the introduction of 1 mg/L of NOM led to a slight decrease in SMX degradation efficiency, with the degradation efficiency dropping to 92%. This suggests that even at low concentrations, NOM can compete with SMX for the available radicals generated through persulfate activation by Fe-BC. When the NOM concentration was elevated to 10 mg/L, a more noticeable effect was observed, with the SMX degradation efficiency decreasing further to 85%. Notably, despite this significant drop, the removal efficiency remained satisfactory. This suggests that, although NOM becomes a more prominent scavenger of radicals at higher concentrations, reducing their availability for SMX degradation, its quenching effect on non-radical Fe(IV) species remains insignificant. Further research is needed to develop strategies to mitigate the adverse effects of NOM on AOP performance, ensuring more effective and reliable contaminant removal in natural water systems.

(4) $$HCO_{3}^{-}+SO_{4}^{•-}\to SO_{4}^{2-}+CO_{3}^{•-}+H^{+}$$

(5) HCO3−+OH•→H2O+CO3•−

## 3. Materials and Methods

### 3.1. Chemicals and Reagents

Sodium persulfate (Na_2_S_2_O_8_, Purity ≥ 99%), Iron(III) chloride hexahydrate (FeCl_3_•6H_2_O, purity ≥ 99%), and sodium hydroxide (NaOH, purity ≥ 98%) were obtained from Sinopharm (Shanghai, China). Additionally, a range of other chemicals were purchased from Sigma-Aldrich (Shanghai, China), including sulfamethoxazole (SMX, purity ≥ 98%), acetaminophen (ACT, 99%), bisphenol A (BPA, purity ≥ 99.8%), benzoic acid (BA, purity >99.5%), phenol (PHE, purity ≥ 99%), nitrobenzene (NB, purity > 99%), *p*-nitrobenzoic acid (*p*-NBA, purity ≥ 99%), dimethyl sulfoxide (DMSO, 98%), methyl phenyl sulfone (PMSO_2_, 98%), phenyl methyl sulfoxide (PMSO, 98%), methanol (MeOH, 99.9%), ethanol (EtOH, 99.9%), furfuryl alcohol (FFA), *p*-benzoquinone (*p*-BQ), sulfuric acid (H_2_SO_4_, 96%), tert-butanol (TBA, purity > 99.5%), acetonitrile, and other necessary chemicals. All chemicals were utilized as received without further purification. Stock and working solutions were prepared using ultrapure water generated by a Milli-Q system (EMD Millipore, Billerica, MA, USA). The pH of the solutions was monitored using an FE28-Standard pH meter (Mettler Toledo, Switzerland, manufactured in Shanghai, China).

### 3.2. Preparation of Fe-BC

In addition to mulberry branches, four types of biomass materials, namely peanut shells, rice straw, and melon seed shells, were selected as the feedstock of carbon. To synthesize Fe-BC composites, 5.0 g of dried biomass powder was thoroughly mixed with an appropriate amount of Fe(NO_3_)_3_•9H_2_O in 100 mL ultrapure water. The mixture underwent magnetic stirring at room temperature for 24 h. Subsequently, the solid precipitate was collected by removing the supernatant liquid, and then the solid was dried overnight in an oven at 80 °C for subsequent use. Finally, the obtained solid precursor was placed in a tube furnace, and the temperature was raised to the set point at a heating rate of 10 °C/min under a N_2_ atmosphere. The carbonization process was carried out for 2 h. The resulting Fe-BC product was then utilized in the subsequent experiments.

### 3.3. Experimental Procedure

The degradation experiments were carried out in a 150 mL conical flask containing 100 mL of reaction solution. The solution included SMX at a concentration of 10 mg/L, Fe-BC primarily prepared from mulberry branches at 800 °C (except when investigating the effects of preparation temperature and different biomass precursors), was utilized as the catalytic material. The Fe-BC was introduced into the solution to achieve a final concentration of 0.2 g/L. PS was introduced to reach concentrations of 2.0 mM (except when exploring the impact of persulfate concentration). The reaction proceeded at 25 °C with continuous stirring. 1 mL of samples was collected at regular intervals (0, 5, 15, 30, 45, and 60 min) and immediately quenched with 200 µL of sodium thiosulfate (Na_2_S_2_O_3_) (50 µM) to terminate the reaction. Subsequently, the samples were filtered through a 0.22 µm membrane. The concentration of SMX in the filtrate was determined by high-performance liquid chromatography (HPLC). The initial pH of the solution was adjusted to the desired value using H_2_SO_4_ or NaOH. It should be noted that the initially measured pH value was obtained after the addition of PS. In all experiments, unless specifically indicated, Fe-BC (Mu) samples obtained from mulberry branches (Mu) were used, which were designated as Fe-BC for short.

### 3.4. Analytical Methods

Quantification of target contaminants was performed on an UltiMate 3000 HPLC system (Thermo Scientific, Waltham, MA, USA) with diode array detection. Chromatographic separation employed an Agilent C18 column (250 mm × 4.6 mm, 5 μm particle size) using either isocratic elution (single-component systems, see Table S1) or optimized gradient conditions (multi-component mixtures, parameters in Table S2). Additionally, reactive species were detected in the water samples using electron paramagnetic resonance spectroscopy (EPR, Bruker A300-10/12, Bruker AXS SE, Karlsruhe, Germany). Specifically, 100 mM 5, 5-dimethyl-1-pyrroline-N-oxide (DMPO) was used for the detection of •OH and SO_4_^•−^, and 100 mM 2,2,6,6-tetra-methylpiperidine (TEMP) was utilized for the detection of singlet oxygen (^1^O_2_).

The crystalline composition of the Fe-BC materials was characterized by utilizing X-ray powder diffraction (XRD) technique employing an instrument from Bruker (D8 ADVANCE, Bruker AXS SE, Karlsruhe, Germany). Additionally, the surface morphology and structural features of the Fe-BC composites were investigated through scanning electron microscopy (SEM), specifically using a Hitachi device (SU8010, Tokyo, Japan).

### 3.5. Steady-State Concentrations of Reactive Species and Their Relative Contribution to SMX Degradation

To quantitatively determine the steady-state concentrations of the key reactive species generated in the Fe–BC/PS system, namely [^•^OH]_ss_, [SO_4_^•−^]_ss_, and [Fe(IV)]_ss_, we applied the competition kinetics method with three widely used probe compounds: nitrobenzene (NB), benzoic acid (BA), and PMSO. These probes have distinct reactivity toward ^•^OH, SO_4_^•−^, and Fe(IV), respectively, and thus allow indirect quantification of the reactive species through kinetic measurements [24]. This method is well established and has been extensively used in related studies [19,24,30,35].

Specifically, the degradation of NB is dominated by ^•^OH, and its kinetic expression can be written as:

(6) d[NB]dt=−kOH•,NB[OH•]ss[NB]

Integration of Equation (1) yields:

(7) ln[NB][NB]0=−kOH•,NB[OH•]sst=−kobs,NBt

where *k_obs,NB_* is the observed rate constant for NB degradation.

Similarly, BA reacts competitively with both ^•^OH and SO_4_^•−^, leading to:

(8) d[BA]dt=−(kOH•,BA[OH•]ss+kSO4•−,BA[SO4•−]ss)[BA]

Integration gives:

(9) ln[BA][BA]0=−(kOH•,BA[OH•]ss+kSO4•−,BA[SO4•−]ss)t=−kobs,BAt

From Equations (7) and (9), the steady-state concentrations can be obtained as follows:

(10) [OH•]ss=kobs,NBkOH•,NB

(11) [SO4•−]ss=kobs,BA−kOH•,BA[OH•]sskSO4•−,BA

The degradation rate of PSMO can be written as Equation (12).

For PMSO, which reacts with ^•^OH, SO_4_^•−^, and Fe(IV), the overall degradation rate can be expressed as:

(12) kobs,PMSO=kOH•,PMSO[OH•]ss+kSO4•−,PMSO[SO4•−]ss+kFe(IV),PMSO[Fe(IV)]ss

Thus, the steady-state concentration of Fe(IV) can be obtained as:

(13) [Fe(IV)]ss=kobs,PMSO−(kOH•,PMSO[OH•]ss+kSO4•−,PMSO[SO4•−]ss)kFe(IV),PMSO

where the $k_{obs,NB}$, $k_{obs,BA}$, $k_{obs,PMSO}$, represent the pseudo-first-order rate constants for the degradation of NB and BA in the Fe-BC/PS system. Meanwhile, kOH•,NB, kOH•,BA, kOH•,PMSO, $k_{SO_{4}^{•-},BA}$ and $k_{SO_{4}^{•-},PMSO}$ are the second-order rate constants representing interactions between ^•^OH and NB, ^•^OH and BA, ^•^OH and PMSO, SO_4_^•−^and BA, as well as SO_4_^•−^ and PMSO.

It is important to note that, according to our previous report [30], the reaction rate constants of singlet oxygen (^1^O_2_) and superoxide radicals (O_2_^•−^) with SMX are very low (Table S3). Therefore, considering the effect of adsorption, the contributions of other species can be neglected in the Fe–BC/PS system, with ^•^OH, SO_4_^•−^, and Fe(IV) identified as the dominant pathways. Accordingly, the removal rate of SMX can be expressed as:

(14) kobs,SMX=kOH•,SMX[OH•]ss+kSO4•−,SMX[SO4•−]ss+kFe(IV),SMX[Fe(IV)]ss+kAdsorption

Here, $k_{obs,SMX}$ and $k_{Adsorption}$ represent the pseudo-first-order rate constants for SMX removal and adsorption in the Fe-BC/PS system, respectively. The second-order rate constants kOH•,SMX, $k_{SO_{4}^{•-},SMX}$ and $k_{Fe(IV),SMX}$ represent the interactions between ^•^OH and SMX, SO_4_^•−^ and SMX, as well as Fe(IV) and SMX.

The relative contribution of each reactive species and adsorption to SMX removal can then be quantitatively determined by:

(15) ROH•=kOH•,SMX[OH•]sskobs,SMX×100%

(16) $$R_{SO_{4}^{•-}}=\frac{k_{SO_{4}^{•-},SMX}{\left[SO_{4}^{•-}\right]}_{ss}}{k_{obs,SMX}}\times 100\%$$

(17) $$R_{Adsorption}=\frac{k_{Adsorption}}{k_{obs,SMX}}\times 100\%$$

(18) RFe(IV)=1−ROH•−RSO4•−−RAdsorption

Through this systematic competition kinetics analysis, the steady-state concentrations of the major reactive species were quantified, and their individual oxidative contributions toward SMX degradation were clearly identified. The results demonstrate that ^•^OH, SO_4_^•−^, and Fe(IV) play the pivotal roles in the transformation of SMX within the Fe–BC/PS system.

## 4. Conclusions

In this study, Fe-BC derived from mulberry branches demonstrated exceptional catalytic performance in activating PS for the efficient degradation of SMX. The synergistic effect of Fe-BC and PS achieved 98% SMX removal within 60 min, significantly outperforming systems with Fe-BC or PS alone. The catalytic activity of Fe-BC was highly dependent on preparation conditions, including calcination temperature, iron loading, and biomass feedstock, with optimal performance observed at 800 °C and 20% Fe loading. Reactive species such as ^•^OH, SO_4_^•−^, and Fe(IV) were identified as key contributors to SMX degradation, with Fe(IV) playing a dominant role due to its selective oxidation of electron-rich functional groups. The degradation efficiency was influenced by water quality parameters, with acidic conditions favoring higher removal rates, while HCO_3_^−^ and NOM exhibited inhibitory effects. The proposed degradation pathways revealed the stepwise oxidation of SMX into smaller intermediates, ultimately mineralizing into CO_2_ and H_2_O. These findings highlight the potential of Fe-BC/PS systems as a sustainable and effective approach for the remediation of sulfonamide antibiotics in aquatic environments, offering insights into optimizing catalyst design and operational parameters for practical applications.

## Data Availability

The data presented in this study are available on request from the corresponding author due to reasonable request.

## Supplementary Materials

The following supporting information can be downloaded at: https://www.mdpi.com/article/10.3390/ijms26209971/s1. References [11,29,31,36,37,38,39,40,41] were cited in Supplementary Materials.
