# Supplementary material for: Iron-Based Biochar for Efficient Persulfate Activation and Sulfamethoxazole Degradation
Source: Int J Mol Sci. 2025 Oct 14;26(20):9971. doi: 10.3390/ijms26209971 (PMC12563551; doi:10.3390/ijms26209971)
Supplement: Supplementary file 1 [file ijms-26-09971-s001.zip › ijms-3894009-supplementary.pdf]

Support information for

Mulberry branch derived iron-based biochar for efficient persulfate activation and sulfamethoxazole degradation

Ying Lu[1,2], Chengdu Qi[3]*, Guilong Peng[2], Yi Gao[4], Ronglong Zhang[4]

[1]*School of Mechatronics and Information Engineering, Chongqing College of Humanities, Science and Technology, Chongqing 401524, China*

[2]*State Key Laboratory of Resource Insects, College of Sericulture, Textile and Biomass Sciences, Southwest University, Chongqing 400715, China*

[3]*School of Environment, Jiangsu Province Engineering Research Center of Environmental Risk Prevention and Emergency Response Technology, Jiangsu Engineering Lab of Water and Soil Eco-remediation, Nanjing Normal University, Nanjing, 210023, China*

[4]*College of Chemistry and Chemical Engineering, Southwest University, Chongqing 400716 China*

Corresponding authors:
E-mail addresses: qichengdu@njnu.edu.cn (Chengdu Qi)

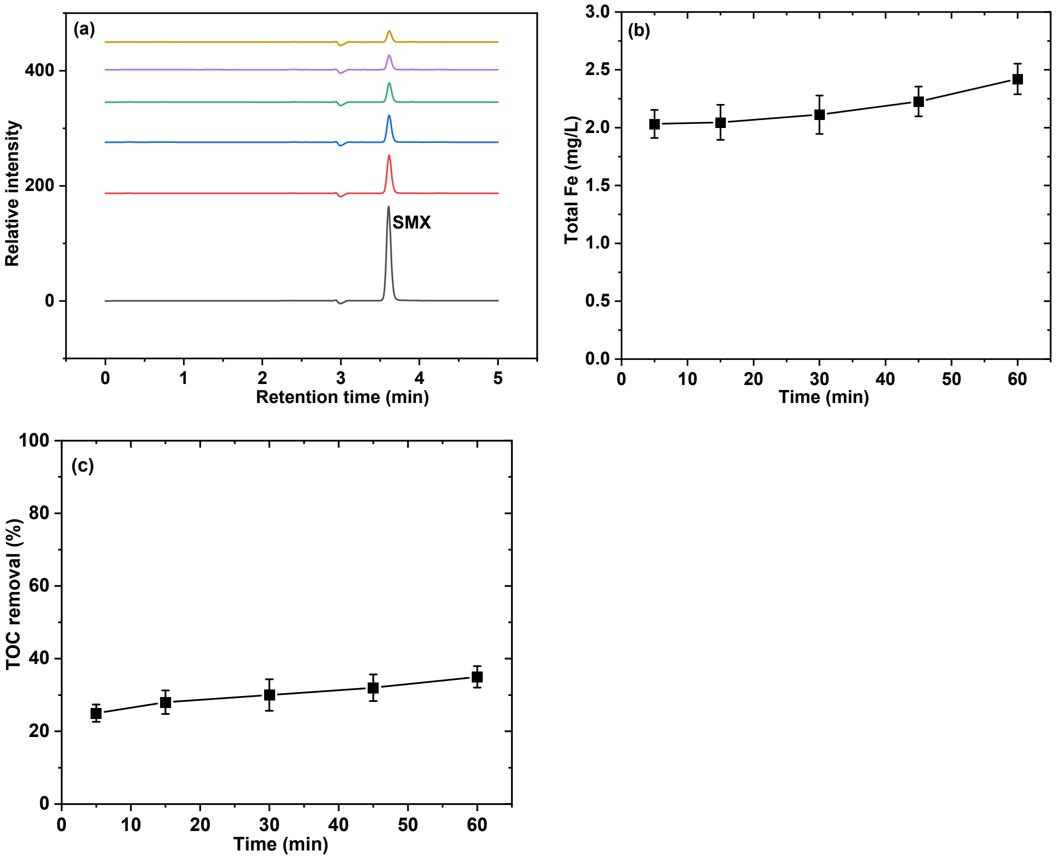

Fig. S1. HPLC chromatogram illustrating the degradation of SMX during treatment with the Fe-BC/PS system (a), the leaching behavior of iron throughout the reaction (b), and the TOC removal. (Conditions: $[Fe\text{-}BC]_0$ = 0.2 g/L, $[PS]_0$ = 2.0 mM, $[SMX]_0$ = 10 mg/L, pH_0 = 3.1).

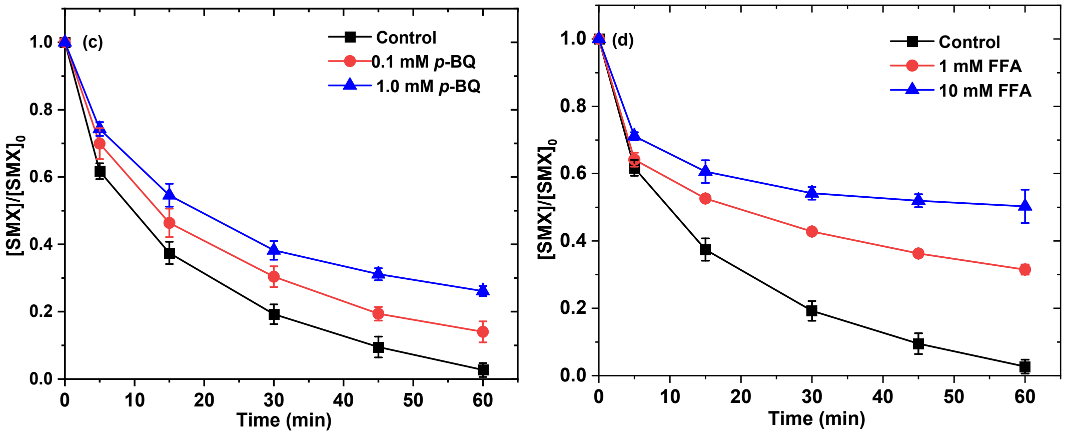

Fig. S2. Effect of (a) *p*-BQ and (b) FFA on SMX removal efficiencies (Conditions: $[Fe\text{-}BC]_0$ = 0.2 g/L, $[PS]_0$ = 2.0 mM, $[SMX]_0$ = 10 mg/L, pH_0 = 3.1).

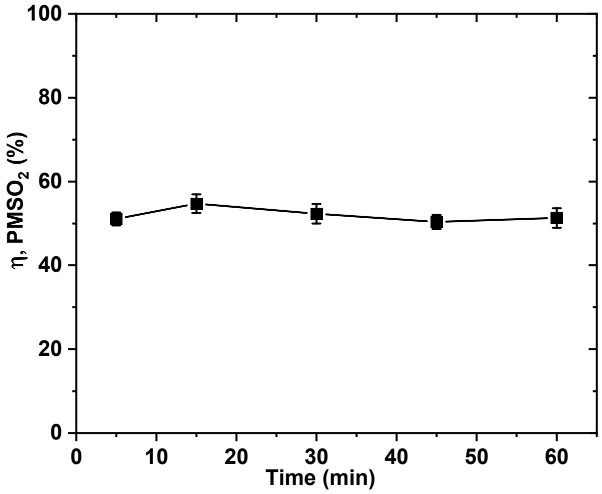

Fig. S4. The yield of $PMSO_2$ during the degradation of PMSO in the Fe-BC/PS system (Conditions: $[Fe\text{-}BC]_0$ = 0.2 g/L, $[PS]_0$ = 2.0 mM, $[PMSO]_0$ = 100 μM, T = 25 °C).

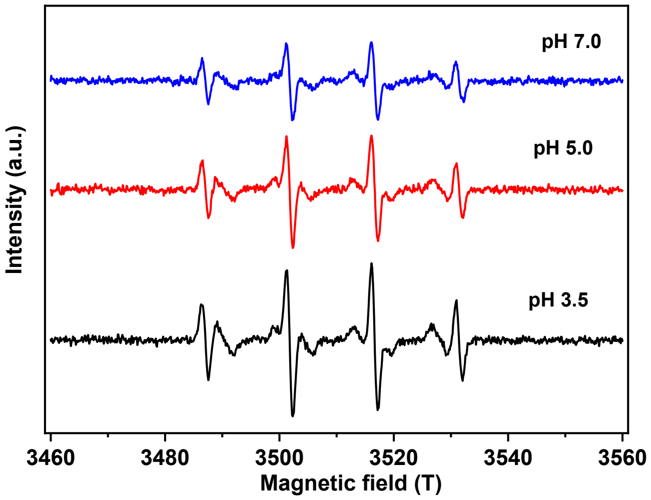

Fig. S5. EPR measurements of $^{\bullet}OH$ and $SO_4^{\bullet-}$ in the Fe-BC/system under different pH values.

Table S1. Details of the mobile phase composition and detection wavelengths for HPLC analysis of organic compounds.

Chemicals	Mobile phase composition	Wavelength (nm)	Flow rate (mL/min)
ACT	Methanol : 0.2% acetic acid = 60 : 40	245	1.0
BPA	Methanol : 0.2% acetic acid = 80 : 20	275	1.0
SMX	Methanol : 0.2% acetic acid = 60 : 40	270	1.0
PHE	Methanol : 0.1% acetic acid = 60 : 40	273	1.0

p-NBA	Methanol : 0.1% acetic acid = 80 : 20	270	1.0
PMSO	Acetonitrile : 0.1% acetic acid = 50 : 50	230	1.0
$PMSO_2$		215	1.0

Table S2. Mobile-phase gradient for the HPLC analysis of a mixture of NB, BA, and PMSO.

Time (min)	Water with 0.2% acetic acid (%)	Methanol (%)	Flow rate (mL/min)
0.0	55	45	1.0
5.0	55	45	1.0
8.0	20	80	1.0
10.0	55	45	1.0

Table S3. Second-order rate constants for the reactions of selected probes, quenchers, and SMX with reactive species in the Fe–BC/PS system.

Target compound (TC)	$k''_{\cdot OH,TC}$ ($M^{-1}s^{-1}$)	$k''_{SO_4^{\cdot -},TC}$ ($M^{-1}s^{-1}$)	$k''_{^1O_2,TC}$ ($M^{-1}s^{-1}$)	$k''_{O_2^{\cdot -},TC}$ ($M^{-1}s^{-1}$)	$k''_{Fe(IV),TC}$ ($M^{-1}s^{-1}$)	Ref.
BA	1.2×10^9	5.9×10^9	- [a]	- [a]	- [a]	[36]
EtOH	2.8×10^9	7.7×10^7	3.8×10^3	- [a]	2.51×10^3	[29, 37]
FFA	1.5×10^{10}	1.3×10^{10}	1.2×10^8	3.5×10^3	- [a]	[29]
NB	3.9×10^9	$< 10^6$	- [a]	- [a]	- [a]	[36]
p-BQ	1.2×10^9	1.0×10^8	6.6×10^7	9.8×10^8	- [a]	[11, 31]
PMSO	3.61×10^9	3.17×10^8	- [a]	- [a]	1.23×10^5 [b]	[38, 39]
$PMSO_2$	3.08×10^9	2.75×10^7	- [a]	- [a]	- [a]	[40]
SMX	8.5×10^9	1.61×10^{10}	2.0×10^4	5.51×10^5	- [a]	[29]
TBA	6.0×10^8	4.0×10^5	3.04×10^3	- [a]	6.0×10^1	[29, 41]

a: not kinetically relevant or unknown

b: measured at pH 1.0

References

[11] Z. Liu, S. Pan, F. Xu, Z. Wang, C. Zhao, X. Xu, B. Gao, Q. Li, Revealing the fundamental role of MoO2 in promoting efficient and stable activation of persulfate by iron carbon based catalysts: Efficient Fe2+/Fe3+ cycling to generate reactive species, Water Res, 225 (2022) 119142.

[29] L. Gao, Y. Guo, J. Zhan, G. Yu, Y. Wang, Assessment of the validity of the quenching method for evaluating the role of reactive species in pollutant abatement during the persulfate-based process, Water Research, 221 (2022) 118730.

[31] Y. Zhao, M. Song, Q. Cao, P. Sun, Y. Chen, F. Meng, The superoxide radicals' production via persulfate activated with CuFe2O4@Biochar composites to promote the redox pairs cycling for efficient degradation of o-nitrochlorobenzene in soil, Journal of Hazardous Materials, 400 (2020) 122887.
[36] Q.-Y. Wu, Z.-W. Yang, Z.-W. Wang, W.-L. Wang, Oxygen doping of cobalt-single-atom coordination enhances peroxymonosulfate activation and high-valent cobalt–oxo species formation, Proceedings of the National Academy of Sciences, 120 (2023) e2219923120.
[37] Y. Zong, X. Guan, J. Xu, Y. Feng, Y. Mao, L. Xu, H. Chu, D. Wu, Unraveling the Overlooked Involvement of High-Valent Cobalt-Oxo Species Generated from the Cobalt(II)-Activated Peroxymonosulfate Process, Environmental Science & Technology, 54 (2020) 16231-16239.
[38] H. Dong, Y. Li, S. Wang, W. Liu, G. Zhou, Y. Xie, X. Guan, Both Fe(IV) and Radicals Are Active Oxidants in the Fe(II)/Peroxydisulfate Process, Environmental Science & Technology Letters, 7 (2020) 219-224.
[39] O. Pestovsky, A. Bakac, Aqueous Ferryl(IV) Ion: Kinetics of Oxygen Atom Transfer To Substrates and Oxo Exchange with Solvent Water, Inorganic Chemistry, 45 (2006) 814-820.
[40] B. Liu, W. Guo, H. Wang, S. Zheng, Q. Si, Q. Zhao, H. Luo, N. Ren, Peroxymonosulfate activation by cobalt(II) for degradation of organic contaminants via high-valent cobalt-oxo and radical species, Journal of Hazardous Materials, 416 (2021) 125679.
[41] Y. Zong, Y. Shao, Y. Zeng, B. Shao, L. Xu, Z. Zhao, W. Liu, D. Wu, Enhanced Oxidation of Organic Contaminants by Iron(II)-Activated Periodate: The Significance of High-Valent Iron–Oxo Species, Environmental Science & Technology, 55 (2021) 7634-7642.
